# Optimization of an in vivo model to study immunity to *Plasmodium falciparum* pre-erythrocytic stages

**DOI:** 10.1186/s12936-019-3055-9

**Published:** 2019-12-18

**Authors:** Yevel Flores-Garcia, Sonia M. Herrera, Hugo Jhun, Daniel W. Pérez-Ramos, C. Richter King, Emily Locke, Ramadevi Raghunandan, Fidel Zavala

**Affiliations:** 1 Department of Molecular Microbiology and Immunology, Malaria Research Institute, Johns Hopkins Bloomberg School of Public Health, Baltimore, MD 21205 USA; 2 PATH Malaria Vaccine Initiative, 455 Massachusetts Avenue, NW, Suite 1000, Washington, DC 20001 USA

**Keywords:** Malaria, Transgenic parasite, Bioluminescence, Vaccine, *Plasmodium falciparum*

## Abstract

**Background:**

The circumsporozoite protein (CSP) of *Plasmodium* is a key surface antigen that induces antibodies and T-cells, conferring immune protection in animal models and humans. However, much of the work on CSP and immunity has been developed based on studies using rodent or non-human primate CSP antigens, which may not be entirely translatable to CSP expressed by human malaria parasites, especially considering the host specificity of the different species.

**Methods:**

Using a genetically engineered strain of *Plasmodium berghei* that expresses luciferase, GFP and the *Plasmodium falciparum* orthologue of CSP, the effect of laboratory preparation, mosquito treatment and mouse factors on sporozoite infectivity was assessed using an in vivo bioluminescence assay on mice. This assay was compared with a PCR-based protection assay using an already described monoclonal antibody that can provide sterile protection against sporozoite challenge.

**Results:**

Bioluminescence assay demonstrated similar detection levels of the quantity and kinetics of liver-stage infection, compared to PCR-based detection. This assay was used to evaluate treatment of sporozoite and delivery method on mouse infectivity, as well as the effects of age, sex and strain of mice. Finally, this assay was used to test the protective capacity of monoclonal antibody AB317; results strongly recapitulate the findings of previous work on this antibody.

**Conclusions:**

The *Pb*GFP-Luc line and in vivo bioluminescence imaging provide highly sensitive read-outs of liver-stage infection in mice, and this method can be useful to reliably evaluate potency of pre-erythrocytic interventions.

## Background

Immune responses to pre-erythrocytic stages of *Plasmodium* induced after immunization with attenuated sporozoites or vaccine candidates confer protective immunity by inhibiting sporozoite infection and the development of liver stages [1, 2]. Work in rodent and non-human primate models of malaria have enabled significant advances in the characterization of immune mechanisms involved in protection against infection by *Plasmodium* sporozoites. The primary target of these immune mechanisms is the circumsporozoite protein (CSP).

Circumsporozoite protein in *Plasmodium berghei* and other *Plasmodium* species that infect animals have significant functional similarity to *Plasmodium falciparum* CSP. However, they have major differences in amino acid sequences. Therefore, studies on the immunogenicity and efficacy of vaccine candidates using non-human plasmodial antigens have limited relevance for the design of human vaccine candidates. Furthermore, because of the strict species-specificity of malaria-causing parasites, the efficacy of vaccines designed for humans cannot be accurately evaluated using animal models; this results in an over-reliance on complex and costly human vaccine trials for initial proof-of-concept data.

The development of transgenic rodent parasites in which the murine CSP is replaced by the *P. falciparum* orthologue helps to overcome some of these limitations. This strategy has previously been used to evaluate the immunogenicity and efficacy of vaccine constructs against *P. falciparum* [3, 4], and *Plasmodium vivax* CSP [5–7], but a detailed analysis of the key features of such an assay has not been reported. Here, in vivo challenge assays are described that use *P. berghei* parasites expressing the *P. falciparum* CSP (3D7) in place of the *P. berghei* CSP; the infectivity of these transgenic parasites is evaluated and quantitative methods are used to assess liver stage development. Critical parameters that affect parasite infection and development in mice are defined, including route of infection, viability and maturity. In addition, host factors that affect the development of liver stages, such as age, sex and genetic background of mice used in these assays are evaluated. Systematic studies using qPCR or other quantitative assays have not addressed all these variables. It is also demonstrated that this in vivo model can be used effectively to determine and compare the protective efficacy of antibodies specific for *P. falciparum* CSP. The aim of this study is to standardize a method that can quantitatively assess sporozoite infectivity and liver stage development under different experimental and physiological conditions. An important objective is also to develop a high throughput assay that may allow the evaluation of multiple immune or pharmacological interventions, permitting a quantitative comparison of reagents that may affect sporozoite infectivity and/or development of the liver stage.

## Methods

### Mice

Six to seven weeks old female mice were used in all experiments, unless otherwise stated for experiments described in Fig. 4c. They were purchased from Charles River Laboratories and maintained at the animal facility of Bloomberg School of Public Health, Johns Hopkins University. All procedures performed in this study were approved by the IACUC Committee under the Protocol MO17H369.

### Parasites

*Plasmodium berghei* used for the transfection express GFP and luciferase and was obtained through BEI Resources, NIAID, NIH: *P. berghei*, strain ANKA 676m1c11, MRA-868, contributed by Chris J Janse and Andrew P Waters [8]. To generate *P. berghei* expressing full-length *P. falciparum* CSP and GFP-luciferase, the same transfection strategy described in detail by Espinosa et al. was followed [4]. Sporozoites were generated by infecting *Anopheles stephensi* mosquitoes by allowing 5-days old mosquitoes to feed on parasite-infected mice at approximately 1–3% parasitaemia. Prior to feeding mosquitoes, the blood of each infected mouse was examined for the presence of gametocyte exflagellation to ensure mosquito infection. This is done by microscopic examination of blood in ookinete medium [7]. After infection, mosquitoes were maintained in an incubator at 19–20 °C. On top of the cages, mosquitoes were supplied with a sterile cotton pad soaked in 10% sucrose, which was changed every 48 h.

Sporozoites were generally harvested at days 20–22 post infection, unless otherwise described. In experiments to evaluate the effect of sporozoite age on infectivity, sporozoites were harvested between 18 and 28 days.

### Bioluminescence measurement

Twenty-four hours after sporozoite injection, mouse abdominal hair was removed using Nair cream. Forty-two hours after injection, the challenged mice were intraperitoneally injected with 100 µL of d-luciferin (30 mg/mL), and then anesthetized in an isoflurane chamber. After mice were immobilized, they were then placed in an IVIS Spectrum imager to evaluate bioluminescence by measuring the radiance for 5 min from the abdomen. For comparison to RT-qPCR quantitation, mouse livers were harvested, and ribosomal RNA was quantified as described previously [9].

### Freezing and thawing parasites

Sporozoites were harvested as described above, counted, re-suspended in FCS, 7% DMSO in FCS or HBSS, and then placed in a pre-cooled NALGENE Cryo 1 **°**C Freezing Container (Cat. No. 5100-0001). The container was placed in a − 80 **°**C freezer and samples were left to freeze overnight. Parasites were then thawed on ice prior to challenge.

### Route of infection

Sporozoites were obtained as described above, and 2000 sporozoites suspended in HBSS-2% FCS and injected through indicated routes. A total volume of 50 µL was injected for intramuscular injections in the leg, subcutaneous injection in the tail base, or subcutaneous injections close to the head. For IV and IP injections, sporozoites were administered in a final volume of 200 µL.

### Mosquito bite challenge

Mice were exposed to the bites of 1–5 infected mosquitoes obtained from a population of mosquitoes that was 70–80% infected as determined by microscopic examination of salivary glands. Mice were anesthetized with 2% Avertin and then placed on top of cages containing mosquitoes for 10 min. Individual mosquitoes used for infection were examined to determine if the mosquitoes successfully fed on mice by assessing the presence of blood in the mosquito midguts. Four days after mosquito bite infection, Giemsa-stained blood smears were examined by light microscopy to look for blood stage infection.

### Protection studies

For both intravenous sporozoite and mosquito bite challenge experiments, mice were first injected in the tail vein with 200 µL of the desired concentration of mAbs, 2 h prior to challenging mice. For intravenous sporozoite challenge experiments, sporozoites were harvested and diluted to 10,000 sporozoites/mL in Hanks balanced salt solution (HBSS) supplemented with 2% fetal calf serum (HBSS-2% FCS), and mice were injected with 2000 sporozoites (200 µL). Bioluminescence data were collected 42 h later. For mosquito bite challenge experiments, mice were anesthetized with 2% Avertin and placed on a cup containing 5 infected mosquitoes for 10 min. Blood stage infection was determined by light microscopy of Giemsa-stained blood films from days 4 to 10.

To test the effect of mAb treatment after infection, mAbs were injected in the tail vein 2 h after sporozoite infection, and bioluminescence data were collected 42 h later.

### Statistical analysis

The data were analysed by using the non-parametric test, the Mann–Whitney test, considering the sample size and lack of evidence for normal distribution.

## Results

### Measuring parasite liver load by RT-qPCR and bioluminescence

**Fig. 1:**
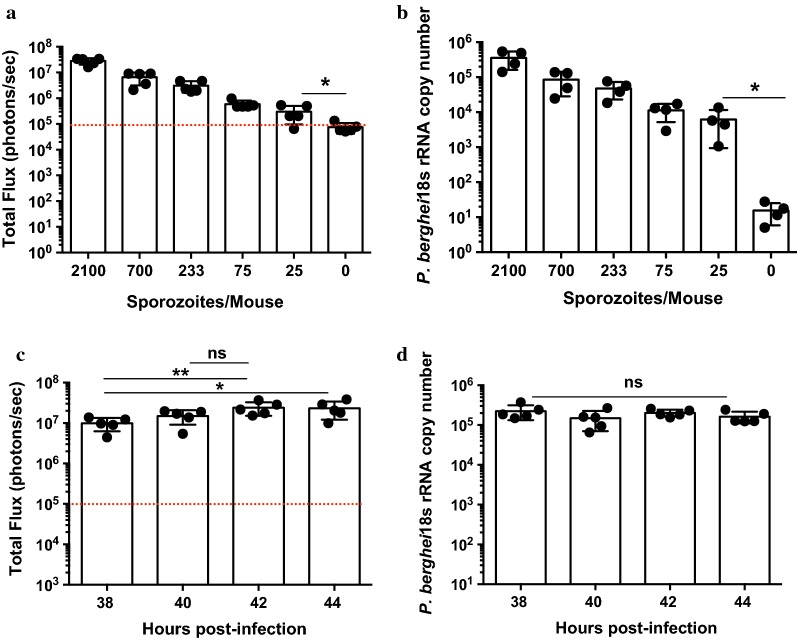
Sensitivity of the bioluminescence assay for liver-stage infection. **a** Luciferin-based luminescence in mice as measured by total flux (photons/second) compared to a titration of the number of sporozoites injected. Naïve non-infected mice treated with d-luciferin served as a control for baseline signal. **b** Measurement of liver burden in the same mice used for luminescence using RT-qPCR *P. berghei* 18s RNA with the same sporozoite titration as in **a**. Both experiments demonstrate dose dependency with a dynamic range of approximately 2 logs. **c** Luminescence measurements of mouse livers at various time points post infection. Data indicate that the peak of infection is approximately 42 h post infection. **d** RT-qPCR measurements of liver infection as in **d,** however when liver burden is measured by RT-qPCR, no statistical significance is observed at different time points tested. *p < 0.05; **p < 0.01, Mann–Whitney test. Dashed lines represent background bioluminescence levels obtained from uninfected mice injected with luciferin

One of the most sensitive and quantitative assays to evaluate the development of *Plasmodium* liver stages is RT-qPCR measuring plasmodial 18S ribosomal RNA in the livers of sporozoite-infected mice [9]. Another assay has been described based on the use of parasite lines expressing luciferase that allows the estimation of parasite burden by measuring parasite specific in vivo bioluminescence in the liver [10]. The sensitivity of both assays was compared in experiments with mice injected intravenously with varying numbers of transgenic *P. berghei* sporozoites expressing luciferase and the *P. falciparum* CSP (Fig. 1a). Forty-two hours after injection of sporozoites, mice were injected intraperitoneally with d-luciferin and then anesthetized with isoflurane. The bioluminescence in the liver was measured 10 min later using an IVIS Spectrum imager (Perkin Elmer). Immediately after imaging, mice were euthanized and their livers excised to extract RNA, to perform RT-qPCR to measure plasmodial 18s rRNA; therefore, the same mice were used to evaluate both bioluminescence and 18s rRNA in the liver. As seen in Fig. 1a, b, both assays gave a good dose–response signal, and were highly sensitive; each detected liver infections that were generated by as few as 25 sporozoites. While the background with bioluminescence is higher, it appears that this measurement in individual mice shows less variability between mice of the same group compared to the RT-qPCR measurements.

To determine the optimal time for the evaluation of parasite load in the liver after sporozoite challenge, groups of mice were injected with 2000 sporozoites and liver bioluminescence was measured at 38, 40, 42, and 44 h after infection as it is well known that parasites are released from hepatocytes 46–48 h after infection [11]. Immediately after imaging, the livers of those mice were excised to measure plasmodial rRNA by RT-qPCR. As shown in Fig. 1c, the bioluminescence measurements indicated significant differences between parasite load measured 38 h after sporozoite injection (9.6 × 10^6^ photons/s) and those recorded at 40 to 44 h (2.3 × 10^7^ photons/s). No statistically significant differences in bioluminescence were found between 40, 42 and 44 h. When estimating the liver parasite load in the same mice using RT-qPCR, (Fig. 1d) differences between groups at these time points did not reach statistical significance. These results confirm that the measurement of bioluminescence gives comparable results with the PCR-based assay.

### Evaluation of sporozoite infectivity

**Fig. 2:**
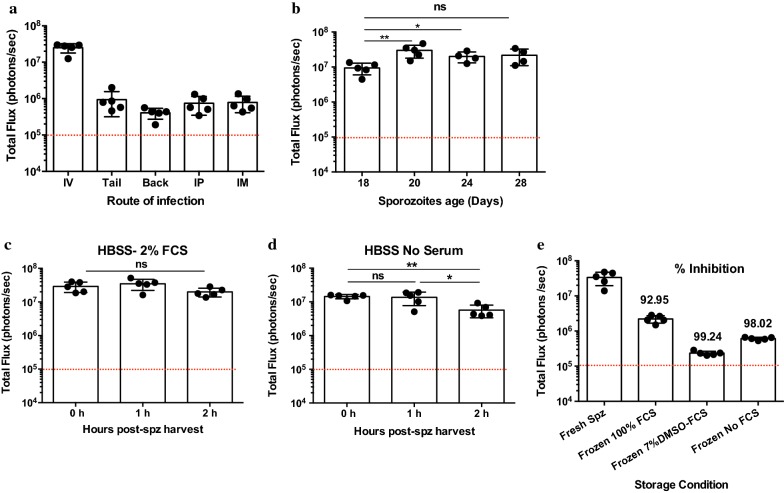
The effect of sporozoite treatment prior to injection. **a** Sporozoite infectivity is highest when delivered intravenously as compared to subcutaneous (tail, back), intraperitoneal and intramuscular injection. **b** Luminescence of mice infected with sporozoites isolated on different days of mosquito infection. Data indicate that sporozoites isolated on day 20 of mosquito infection are most infective. Luminescence readings were taken 42 h after mouse infection. The red dotted line indicates the background signal as determined in Fig. 1a. **b**, **c** Mouse luminescence of sporozoites dissected in HBSS supplemented with 2% FCS (**b**) or without FCS (**c**) and injected at various times after dissection. Dissection in serum provides greater sporozoite infectivity than dissection without serum. Dissecting without serum also results in a time-dependent loss of infectivity. **d** Comparison of infectivity of fresh sporozoites to sporozoites frozen under various different media compositions. All frozen sporozoites are > 90% less infective than freshly isolated sporozoites. *p < 0.05; **p < 0.01, Mann–Whitney test. Dashed lines represent background bioluminescence levels obtained from uninfected mice injected with luciferin

Using the bioluminescence assay, the infectivity of the same number of sporozoites was compared by injection through different routes. As shown in Fig. 2a, it is clear that maximum infectivity was achieved when parasites were injected intravenously. In contrast, subcutaneous injection in the tail and the back, intramuscular or intraperitoneal routes resulted in a major reduction (90% or greater) in liver burden compared to IV infection. To evaluate the effect that sporozoite age may have on infectivity, 2000 sporozoites were obtained from mosquito salivary glands and injected IV at different times after infection. The results shown in Fig. 2b indicate that sporozoites obtained 18 days after mosquito blood feeding are less infective (Geomean 8.8 × 10^6^ photons/s) than sporozoites obtained at day 20 or later (Geomean 2.7 × 10^7^ photons/s). Sporozoites obtained 28 days after infection are fully infectious, even though at this time, most mosquitoes have died, and the salivary glands have reduced numbers of sporozoites.

Next, the viability of sporozoites after purification from salivary glands was evaluated. As shown in Fig. 2c, sporozoites maintained at 4 **°**C in HBSS supplemented with 2% fetal calf serum (2% FCS-HBSS), maintained their capacity to infect mice after 2 h. When sporozoites were maintained in HBSS without FCS for 2 h, there was a marked loss of infectivity of approximately 60% with time (Geomean 5.3 × 10^6^ photons/s vs 1.4 × 10^7^ photons/s), as earlier studies have shown [12]. Freezing sporozoites using different approaches [13] also had a major effect on their infectivity, as the results shown in Fig. 2e indicate that freezing parasites severely reduces infectivity by nearly 2 logs.

**Fig. 3:**
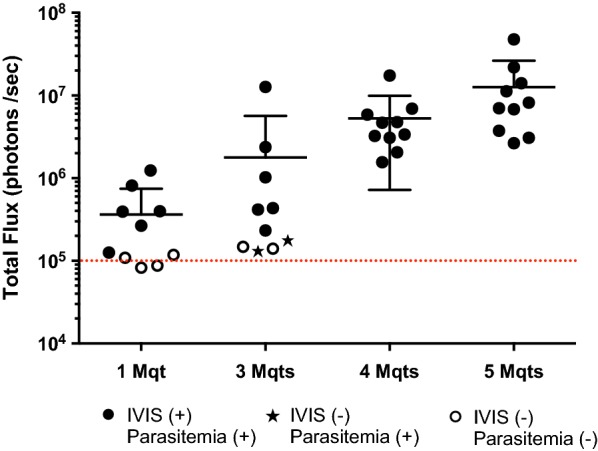
Mosquito bite challenge. Challenge by bites from 1 or 3 infectious mosquitoes does not guarantee successful infection, while bites from 4 or greater mosquitoes do guarantee successful infection. Filled circle indicates mice that were IVIS positive and positive for blood stage infection; open circle indicates mice that were negative for both IVIS imaging and blood stage infection; filled star indicates mice that were negative for IVIS imaging, but positive for blood stage infection. Dashed lines represent background bioluminescence levels obtained from uninfected mice injected with luciferin

Mice were also infected by exposure to the bites of *An. stephensi* infected with transgenic parasites to determine the minimum number of infectious mosquitoes that are required to generate a reliable blood stage infection. It was found that the exposure to the bites of 4–5 infected mosquitoes generates blood stage infection in 100% of mice as detected by bioluminescence imaging and confirmed by the presence of infected erythrocytes by blood smear 4–6 days after exposure to mosquito bites (Fig. 3). When mice were exposed to the bites of 1 or 3 infected mosquitoes, many of the mice did not become infected. In some mice, the infection was detected only in the blood, indicating that the infectious inoculum injected by mosquitoes was too small to generate detectable liver infection.

### Host factors affecting liver stage development

**Fig. 4:**
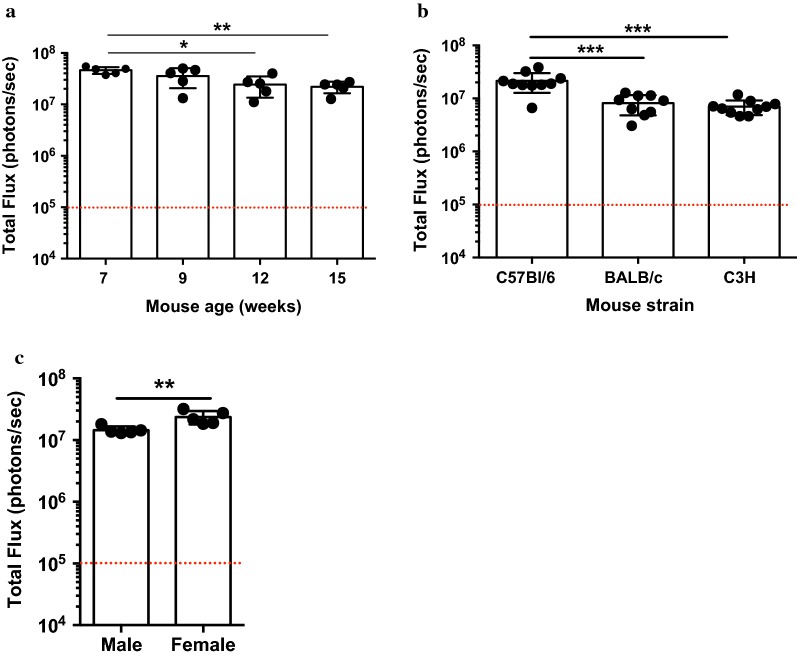
The effect of mouse variables on sporozoite infectivity. **a** Luminescence of C57Bl/6 mice of different ages challenged with the same number of sporozoites. Mice challenged at 7 weeks of age were most susceptible to infection, and this susceptibility decreases with age, with statistical significance at days 12 and 15. **b** Luminescence data of C57Bl/6, BALB/c, and C3H mice infected with sporozoites. C57Bl/6 mice are the most susceptible of the three strains. **c** Comparison of infectivity of male and female C57Bl/6 mice. Female mice demonstrate higher susceptibility to infection than males. *p < 0.05; **p < 0.01; ***p < 0.0005, Mann–Whitney test. Dashed lines represent background bioluminescence levels obtained from uninfected mice injected with luciferin

The age of infected mice impacts parasite development. After infecting mice between 7 and 15 weeks old, using 5 mice per group, an age-dependent reduction in infectivity was observed (Fig. 4a). There is a statistically significant reduction of 51% in 12 weeks old mice (Geomean 2.2 × 10^7^ photons/s) and 53% in 15 weeks old mice (Geomean 2.1 × 10^7^ photons/s) compared to the 7 weeks old cohort (Geomean 4.5 × 10^7^ photons/s).

The genetic background of the mice is also a factor that influences parasite development. C57Bl/6 mice are most sensitive to parasite infection (1.0 × 10^7^ photons/s) as compared to BALB/c and C3H mice, whose liver parasite load were reduced by approximately 65% (Geomean 7.4 × 10^6^ photons/s and 6.7 × 10^6^ photons/s, respectively) (Fig. 4b). In order to evaluate if the sex of the mouse affects sporozoite infectivity, male and female mice C57Bl/6 of the same age were infected with 2000 sporozoites. Figure 4c shows that development of liver stages is 37.7% lower in males (1.4 × 10^7^ photons/s) compared to females (2.3 × 10^7^ photons/s).

### Protective activity of anti-sporozoite antibodies

This in vivo model using transgenic parasites can be a useful tool for preclinical studies aimed at evaluating and characterizing protective immune mechanisms against *P. falciparum* sporozoites. Using this assay, the inhibition of development of liver stages conferred by passive transfer of human monoclonal antibodies (mAbs) was evaluated.

**Fig. 5:**
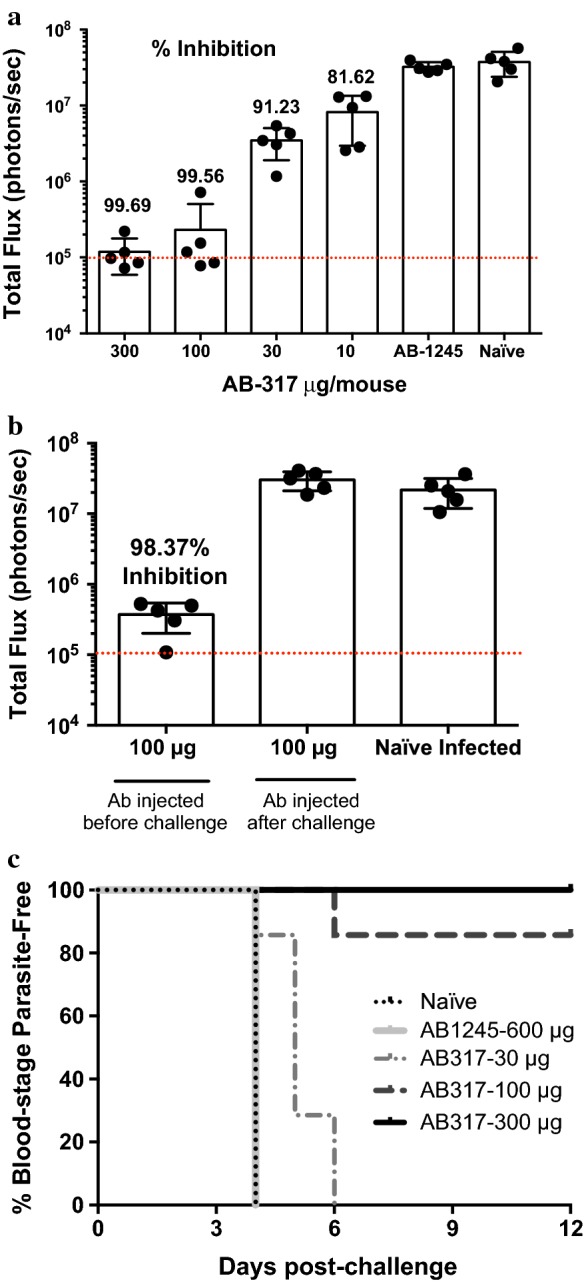
Using IVIS imaging for protection models. **a** Monoclonal antibody protection prior to infection. Treatment with 300 μg of AB317 before IV injection of parasites, confers > 99% inhibition of liver stage development as measured by IVIS. There is dose dependent reduction of liver stage development as AB317 treatment decreases. The numbers on top of each histogram represents the percent inhibition compared to the parasite load measured in naïve mice. **b** Treatment with AB-317 before and after sporozoite challenge. Treatment before challenge inhibits liver stage development, while treatment after challenge has no effect. **c** Sterile immunity, as assessed by lack of blood parasitaemia, after mAb treatment followed by exposure to mosquito bites. Kaplan–Meier curves demonstrate dose-dependent protection by AB317, with complete protection with 300 μg. 1245, a mAb directed against an irrelevant antigen, is used as a negative control for mAb treatment. Dashed lines represent background bioluminescence levels obtained from uninfected mice injected with luciferin

Monoclonal antibody AB317 is known to recognize the NANP repeat domain of *P. falciparum* CSP [14]. Prior to sporozoite challenge, mice were injected with 300, 100, 30 or 10 µg of AB317. This resulted in a potent, dose-dependent reduction of liver infection Fig. 5a and is in agreement with previous experiments (Fig. 5a) [14]. Importantly, this effect was only observed when the antibody injection was performed before infection with sporozoites, as passive transfer of mAb 2 h after parasite infection had no effect on the development of liver stages (Fig. 5b). This indicates that the anti-parasite mechanism of this mAb is by inhibition of sporozoite invasion of hepatocytes and does not affect the development of the parasite once it is inside the host cell.

A critical feature of protective immunity is the ability of antibodies to confer sterile immunity when evaluated in conditions resembling those found in areas of natural parasite transmission. Using the mosquito bite model of infection, the capacity of AB317 to confer sterile immunity was evaluated. As shown in Fig. 5c, mice receiving 300 µg of AB317 followed by exposure to the bites of five infected mosquitoes were completely protected from progression to blood-stage infection. Similar to the observations seen in measuring liver burden (Fig. 5a), this protection is dose dependent.

## Discussion

The rapid and accurate assessment of sporozoite infectivity of the liver is critical for the development of new immunological or pharmacological interventions aimed at blocking this stage of the parasite life cycle. The mouse challenge model reported here allows the comparative screening of antibodies administered through passive or active immunization in mice. A transgenic *P. berghei* strain that expresses *P. falciparum* CSP was generated. As determined by immunofluorescence and ELISA assays, the transgenic sporozoites express nearly identical amounts of CSP compared to *P. falciparum* sporozoites, and have identical infectivity to wild type *P. berghei* ANKA parasites (Additional file 1: Figure S1C). Critical parameters were investigating regarding the method of detection of liver infection, sporozoite preparation, and host factors and each demonstrated a dose response range for inhibition of infection.

The RT-qPCR assay is based on the measurement of plasmodial 18s rRNA in liver, while bioluminescence imaging measures sporozoite-induced gene expression in the liver as luciferase activity. It was found that both assays can efficiently detect liver infections generated by as few as 25 sporozoites. An important factor favouring the bioluminescence approach is the complexity of the RT-qPCR methodology that involves a number of different steps, such as RNA purification from livers, generation of cDNA, and PCR amplification, all of which are likely to accumulate experimental errors and increase variability. In contrast, the assessment of liver burden by bioluminescence is a procedure made in live mice, without manipulation of the parasites, is sensitive, reproducible and much easier to perform, allowing increased throughput without significant loss of specificity or sensitivity (Additional file 1: Figure S1A, B; Table S1).

The bioluminescence-imaging assay enabled definition of basic assay features that influence detection of sporozoite liver infection. Using the bioluminescence assay, it was possible to discern differences in sporozoite infectivity due to route of infection, sporozoite age and treatment, as well as host factors, including mouse age, strain, and sex.

Importantly, it was determined that mice could be reproducibly infected by exposure to bites of 4–5 infected mosquitoes, resulting in clearly measurable levels of parasite liver load. In contrast, the exposure of mice to the bites of 1–3 mosquitoes does not reliably result in blood stage infection, even though it was confirmed that those mosquitoes were infected by the presence of sporozoites in their salivary glands. These results are consistent with prior studies, which indicate that the bites from single infected mosquitoes have a 30–70% probability to induce blood stage parasitaemia and that this may depend on sporozoite load in salivary glands [15, 16].

This assay was used to evaluate the protective capacity of antibodies specific for *P. falciparum* CSP and found a clear dose response in conferring inhibition of liver stage development. Since this inhibition is strictly dependent on the injection of antibodies before parasite challenge, we conclude that the protective activity of antibody AB317 results from neutralization of sporozoites before invading hepatocytes. Importantly, this antibody does not inhibit the infectivity of wild type *P. berghei* sporozoites (Additional file 1: Figure S1D). The lack of effect of AB1245 is expected, as this is an irrelevant antibody that does not recognize sporozoites. It was determined that this in vivo model could also be used to assess the capacity of these of antibodies to confer sterile immunity after challenge of mice through the bites of infected mosquitoes. When challenging mice by exposure to the bites of 5 infected mosquitoes, a dose-dependent protection was found, with 100% protection conferred with mice receiving 300 µg of AB317.

## Conclusions

A bioluminescence assay that provides data similar to RT-qPCR assessment of *Plasmodium* liver-stage infection in mice, which is more rapid than RT-qPCR and can use fewer mice in order to assess pre-erythrocytic interventions, was developed. This in vivo model should greatly help to evaluate the efficacy of multiple CSP-based vaccine constructs and immunization regimens as a screen to select the most effective and efficient vaccine candidates prior to evaluation in controlled human malaria infection trials. It should also be helpful to define the efficacy of passively transferred mAbs, including those obtained from immunized, challenged and protected (or non-protected) individuals. These mAbs can provide valuable information on the precise conformation of epitopes recognized by protective antibodies, thus eliminating unnecessary or inhibitory epitopes and guiding vaccine design. In addition, this method offers the advantage of measuring both liver infection and blood-stage infection in the same mouse, reducing the number of mice required for measuring both liver load and the pre-patency period, allowing studies to more precisely link the two phenomena. Finally, this tool can be modified to evaluate other human plasmodial antigens of interest. Recent surface proteome work in *P. falciparum* [17] and *P. vivax* [18] has identified other possible vaccine candidates. Transgenic *P. berghei* parasites expressing other plasmodial antigens could be generated and this model could interrogate whether or not these surface proteins may have potential as vaccine candidates.

## Supplementary information

**Additional file 1: Table S1**; **Figure S1. (A)** Analysis of CSP expression by immunofluorescence. Sporozoites of *P. falciparum NF54* strain and transgenic *P. berghei* sporozoites expressing the *P. falciparum* CSP (*PbPf* SPZ) were dissected from mosquito salivary glands, placed in wells of immunofluorescence slides, air-dried fixed, and incubated with AB317 specific for PfCSP (5 ng/mL). Anti-human IgG labelled with AF594 was used as a secondary antibody. **(B)** ELISA with *P. falciparum* and *P. berghei* sporozoites expressing the *P*. *falciparum* CSP was performed by attaching 5,000 sporozoites of each species onto microtiter plates, as described previously [19]. ELISA was performed by incubating in each well with different amounts of AB317, which recognizes *P. falciparum* CSP. Results show very similar binding curves for *P. falciparum* and transgenic *P. berghei* sporozoites, indicating comparable levels of expression between the two parasite lines. **(C)** Sporozoite infectivity in C57Bl/6 mice. Mice were inoculated intravenously with 2,000 wild type *P. berghei* or transgenic *PbPf* SPZ sporozoites. 42 h later parasite liver burden was measured by bioluminescence. Both parasites showed the same infectivity. 5 mice per group were used and the results show the bioluminescence mean ± standard deviation for each group. **(D)** Graphs show the effect of anti-*P. falciparum* CSP monoclonal antibody AB317 which inhibits infectivity of transgenic parasites, but has no effect on the infectivity of wild type *P. berghei* sporozoites.

## Data Availability

All data generated or analysed during this study are included in this published article.

## Abbreviations

CSP: circumsporozoite protein
FCS: fetal calf serum
GFP: green fluorescent protein
HBSS: Hank’s Balanced Salt Solution
Luc: luciferase
NHP: non-human primate
RT-qPCR: reverse transcriptase quantitative polymerase chain reaction
mAb: monoclonal antibody
