# Supplementary material for: Optimization of an in vivo model to study immunity to Plasmodium falciparum pre-erythrocytic stages
Source: Malar J. 2019 Dec 18;18:426. doi: 10.1186/s12936-019-3055-9 (PMC6918627; doi:10.1186/s12936-019-3055-9)
Supplement: Supplementary file 1 — Additional file 1: Table S1; Figure S1. (A) Analysis of CSP expression by immunofluorescence. Sporozoites of P. falciparum NF54 strain and transgenic P. berghei sporozoites expressing the P. falciparum CSP (PbPf SPZ) were dissected from mosquito salivary glands, placed in wells of immunofluorescence slides, air-dried fixed, and incubated with AB317 specific for PfCSP (5 ng/mL). Anti-human IgG labelled with AF594 was used as a secondary antibody. (B) ELISA with P. falciparum and P. berghei sporozoites expressing the P. falciparum CSP was performed by attaching 5,000 sporozoites of each species onto microtiter plates, as described previously [19]. ELISA was performed by incubating in each well with different amounts of AB317, which recognizes P. falciparum CSP. Results show very similar binding curves for P. falciparum and transgenic P. berghei sporozoites, indicating comparable levels of expression between the two parasite lines. (C) Sporozoite infectivity in C57Bl/6 mice. Mice were inoculated intravenously with 2,000 wild type P. berghei or transgenic PbPf SPZ sporozoites. 42 h later parasite liver burden was measured by bioluminescence. Both parasites showed the same infectivity. 5 mice per group were used and the results show the bioluminescence mean ± standard deviation for each group. (D) Graphs show the effect of anti-P. falciparum CSP monoclonal antibody AB317 which inhibits infectivity of transgenic parasites, but has no effect on the infectivity of wild type P. berghei sporozoites. [file 12936_2019_3055_MOESM1_ESM.pptx]

## Slide 1
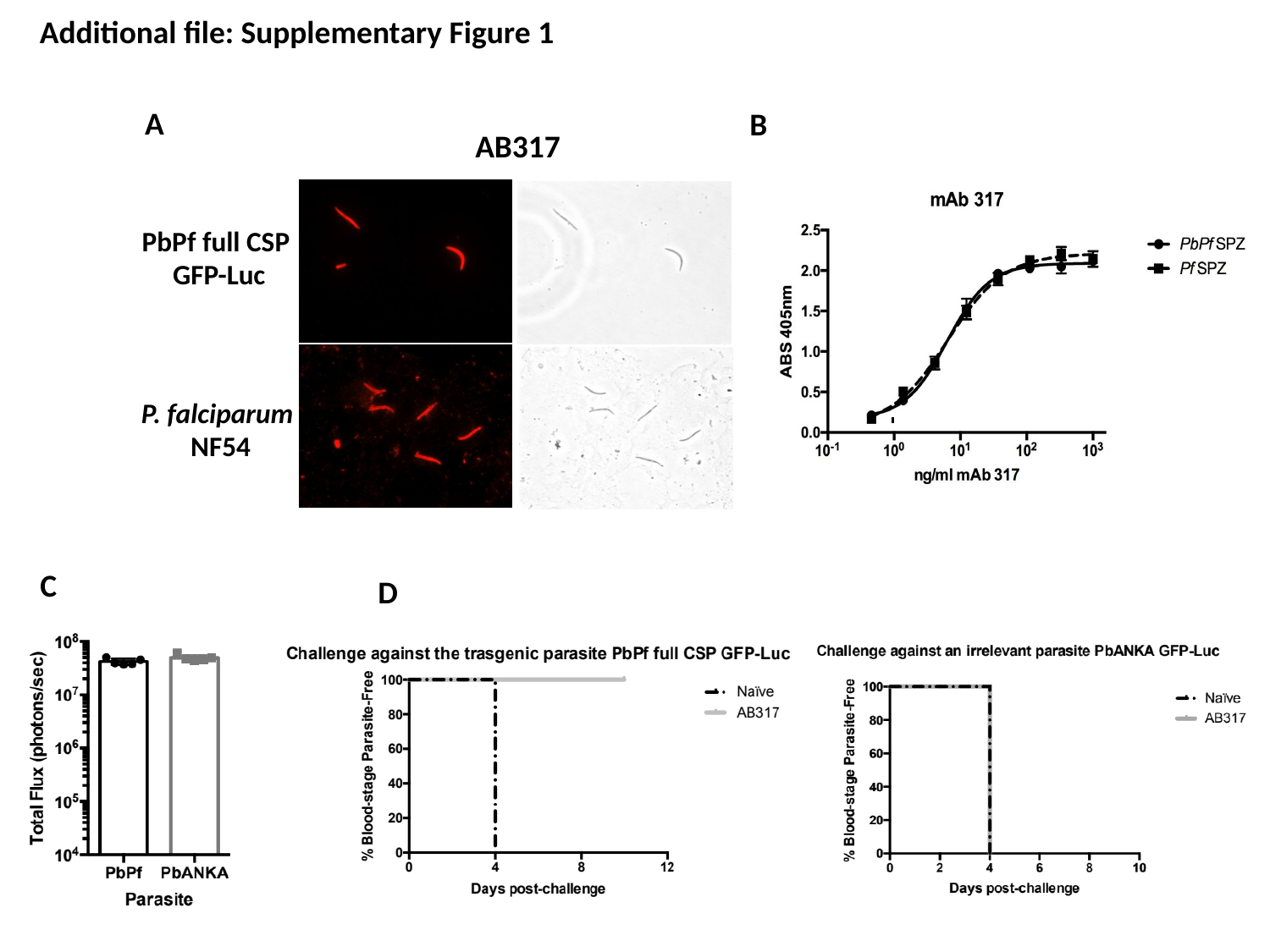

Additional file: Supplementary Figure 1
A
AB317
PbPf full CSP
GFP-Luc
P. falciparum
 NF54
B
C
D

## Slide 2
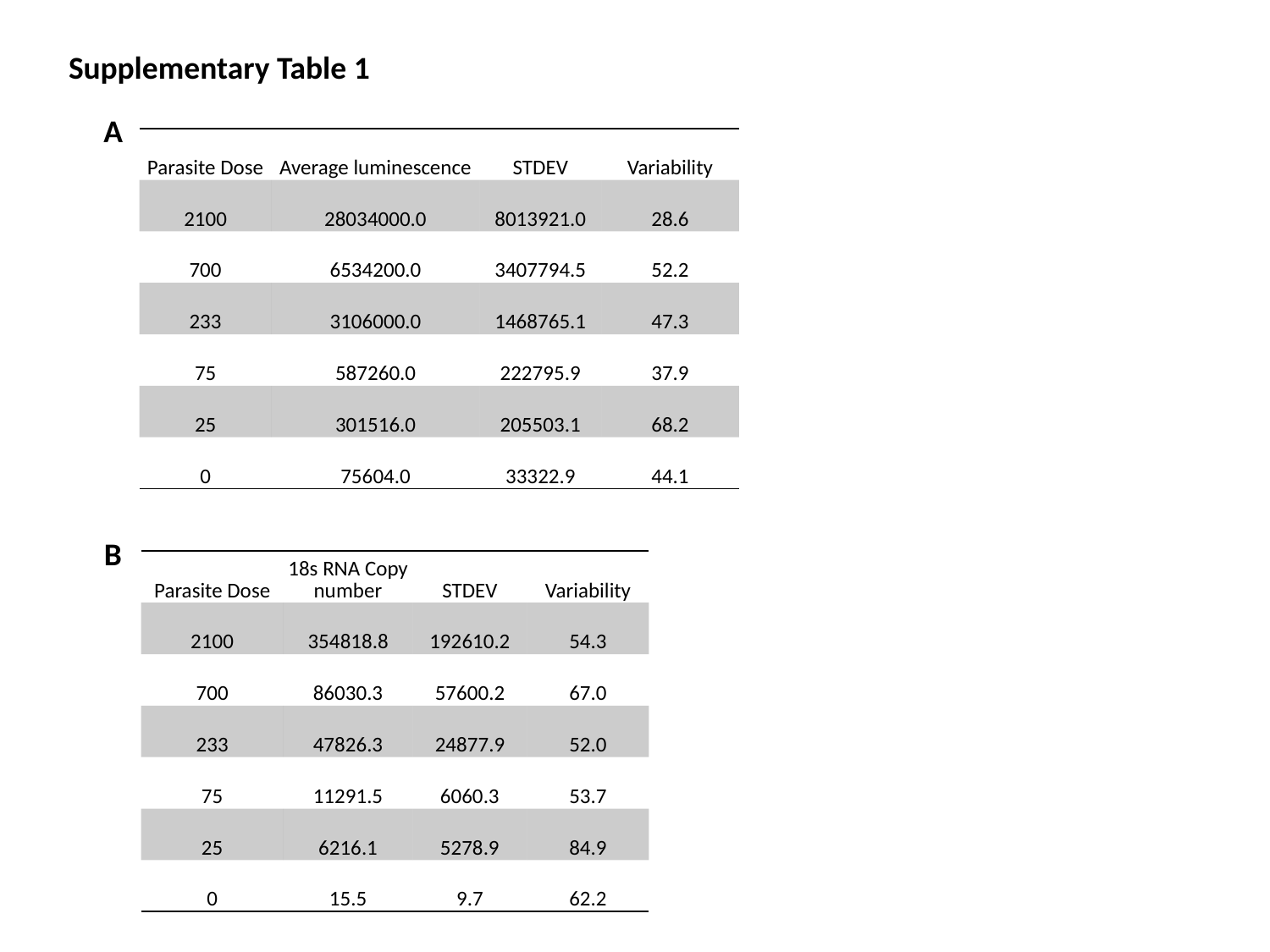

Supplementary Table 1
A
| Parasite Dose | Average luminescence | STDEV | Variability |
| --- | --- | --- | --- |
| 2100 | 28034000.0 | 8013921.0 | 28.6 |
| 700 | 6534200.0 | 3407794.5 | 52.2 |
| 233 | 3106000.0 | 1468765.1 | 47.3 |
| 75 | 587260.0 | 222795.9 | 37.9 |
| 25 | 301516.0 | 205503.1 | 68.2 |
| 0 | 75604.0 | 33322.9 | 44.1 |
B
| Parasite Dose | 18s RNA Copy number | STDEV | Variability |
| --- | --- | --- | --- |
| 2100 | 354818.8 | 192610.2 | 54.3 |
| 700 | 86030.3 | 57600.2 | 67.0 |
| 233 | 47826.3 | 24877.9 | 52.0 |
| 75 | 11291.5 | 6060.3 | 53.7 |
| 25 | 6216.1 | 5278.9 | 84.9 |
| 0 | 15.5 | 9.7 | 62.2 |
